# A novel BRAF::PTPRN2 fusion in meningioma: a case report

**DOI:** 10.1186/s40478-023-01668-w

**Published:** 2023-12-08

**Authors:** Nishanth S. Sadagopan, Khizar R. Nandoliya, Mark W. Youngblood, Craig M. Horbinski, Jared T. Ahrendsen, Stephen T. Magill

**Affiliations:** 1 Department of Neurological Surgery, Northwestern University Feinberg School of Medicine, 676 N. St. Clair Street, Suite 2210, Chicago, IL 60611 USA; 2 Department of Pathology, Northwestern University Feinberg School of Medicine, 303 E. Chicago Avenue, Ward 3-140, Chicago, IL 60611 USA

**Keywords:** BRAF, PTPRN2, Gene Fusion, Meningioma, Chromothripsis, Chromosome 7q

## Abstract

Gene fusion events have been linked to oncogenesis in many cancers. However, gene fusions in meningioma are understudied compared to somatic mutations, chromosomal gains/losses, and epigenetic changes. Fusions involving B-raf proto-oncogene, serine/threonine kinase (*BRAF*) are subtypes of oncogenic *BRAF* genetic abnormalities that have been reported in certain cases of brain tumors, such as pilocytic astrocytomas. However, *BRAF* fusions have not been recognized in meningioma. We present the case of an adult female presenting with episodic partial seizures characterized by déjà vu, confusion, and cognitive changes. Brain imaging revealed a cavernous sinus and sphenoid wing mass and she underwent resection. Histopathology revealed a World Health Organization (WHO) grade 1 meningioma. Genetic profiling with next generation sequencing and microarray analysis revealed an in-frame *BRAF::PTPRN2* fusion affecting the BRAF kinase domain as well as chromothripsis of chromosome 7q resulting in multiple segmental gains and losses including amplifications of cyclin dependent kinase 6 (*CDK6*), tyrosine protein-kinase Met (*MET*), and smoothened (*SMO*). Elevated pERK staining in tumor cells provided evidence of activated mitogen-activated protein kinase (*MAPK*) signaling. This report raises the possibility that gene fusion events may be involved in meningioma pathogenesis and warrant further investigation.

## Introduction

Meningiomas are the most common primary brain tumors comprising one third of all central nervous system tumors [1]. The World Health Organization (WHO) groups meningiomas into three grades based on their histopathological and molecular characteristics. Distinguishing between grades of meningiomas has important implications for treatment, management, and prognosis [2]. Approximately 80% of meningiomas are WHO grade 1 and can be managed with surgery or stereotactic radiosurgery (SRS) with low recurrence risk, while about 20% are atypical or anaplastic subtypes (WHO grade 2 and 3, respectively) with associated increased recurrence and malignant potential [3–5].

While the WHO grading provides a framework to manage and prognosticate meningioma behavior, some meningiomas may not behave according to their WHO grade [6]. Molecular and genetic analysis of meningiomas through next generation sequencing (NGS) and DNA methylation profiling are increasingly used to refine our understanding of meningioma recurrence risk [7–13]. Recent studies using RNA sequencing and DNA methylation profiling have shown that meningiomas can be categorized according to their methylation or gene expression patterns [12, 14, 15]. NGS has led to the identification of novel driver gene mutations in meningioma in addition to *NF2*, including *SMO, PI3KCA, TRAF7, KLF4, AKT1, POLR2A*, and *SMARCE1* [7, 16–20]. Discovery of driver mutations can offer additional routes of personalized therapy for surgically challenging or recurrent meningiomas, as in the Alliance A071401 Clinical Trial [21–23]. Identification of genetic markers in meningioma such as *TERT* and *CDKN2A* provides insight into prognosis and potential recurrence [24, 25]. Molecular sequencing has also revealed potential therapeutic targets in meningioma including *SMO, AKT/PI3K*, and *BAP-1* [26]. Incorporating molecular and genetic insights with histopathological analysis is advancing characterization and treatment of meningioma; however, limited studies have investigated the role of gene fusions in meningioma.

Here, we report the case of an adult female presenting with episodic partial seizures who was found to have a WHO grade 1 left cavernous sinus/sphenoid wing meningioma. Genetic profiling of her resected tumor with NGS revealed no pathologic mutations. Gene fusion panel identified a 7q34 B-raf proto-oncogene, serine/threonine kinase (*BRAF*) fusion with a 7q36.3 protein tyrosine phosphatase receptor type N2 (*PTPRN2*). Copy number profiling revealed chromothripsis of chromosome 7q resulting in multiple segmental gains and losses including amplifications of 7q21.2–7q22.1 containing cyclin dependent kinase 6 (*CDK6*), 7q31.1–7q31.31 containing tyrosine protein-kinase Met (*MET*), 7q31.33 - q32.1 containing smoothened (*SMO*), as well as amplifications of both 7q33, 7q36.1 and 7q36.3. Additional immunostaining revealed pERK positivity in tumor cells, indicating increased mitogen-activated protein kinase (*MAPK*) pathway activation. Methylation profiling matched to a Merlin-intact meningioma, consistent with the copy number profile of this tumor. To our knowledge, this is the first *BRAF* fusion reported in meningioma. Future investigations into the significance of *BRAF* fusions in meningioma may help select good candidates for targeted anti-*BRAF* therapy.

## Case presentation

**Fig. 1:**
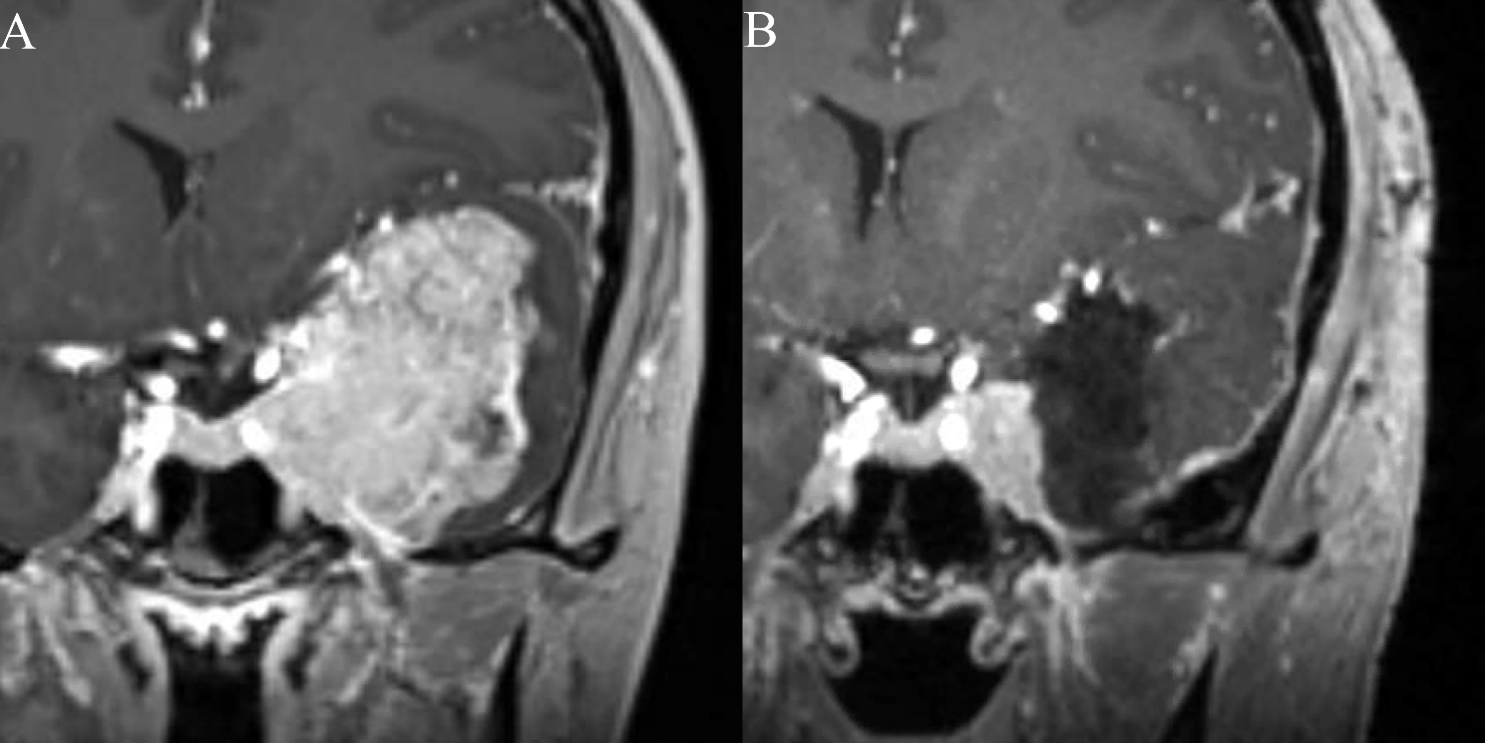
**A**) Pre-operative MRI showing a large middle cranial fossa mass with surrounding edema, significant compression of the left temporal lobe and effacement of the lateral ventricle. **B**) Post-operative MRI showing residual meningioma in the cavernous sinus following planned subtotal resection to preserve neurological function

The patient was a healthy 30-year-old right-handed female with no significant past medical history who presented with one month of multiple acute episodes of déjà vu, phantosmia (odorant perceived in the absence of stimulus), and Broca’s aphasia followed by sensations of panic and dizziness. She occasionally had difficulty finding words for a short period after the episodes. She had some mild decreased left facial sensation. The patient was referred to the epilepsy team for work up of her partial seizures. Electroencephalogram (EEG) did not show epileptiform activity. Brain magnetic resonance imaging (MRI) revealed an avidly enhancing 5.9 × 4.8 cm left cavernous sinus and sphenoid wing mass with cerebral edema, significant compression of the left temporal lobe and left frontal lobe, 3 mm midline shift **(**Fig. 1a**)**, and invasion into the left cavernous sinus, left anterior clinoid, and the left superior orbital fissure. Edema of the left hippocampus and temporal gyri was present on brain MRI. The patient was started on 500 mg of Levetiracetam twice a day for management of the partial seizures.

Given the size and location of the mass as well as the patient’s young age, the patient underwent a left cranio-orbital craniotomy with planned subtotal resection (Fig. 1b),  with intentional residual left inside the cavernous sinus to preserve cranial nerve function. The procedure was completed without intraoperative complication.

**Fig. 2:**
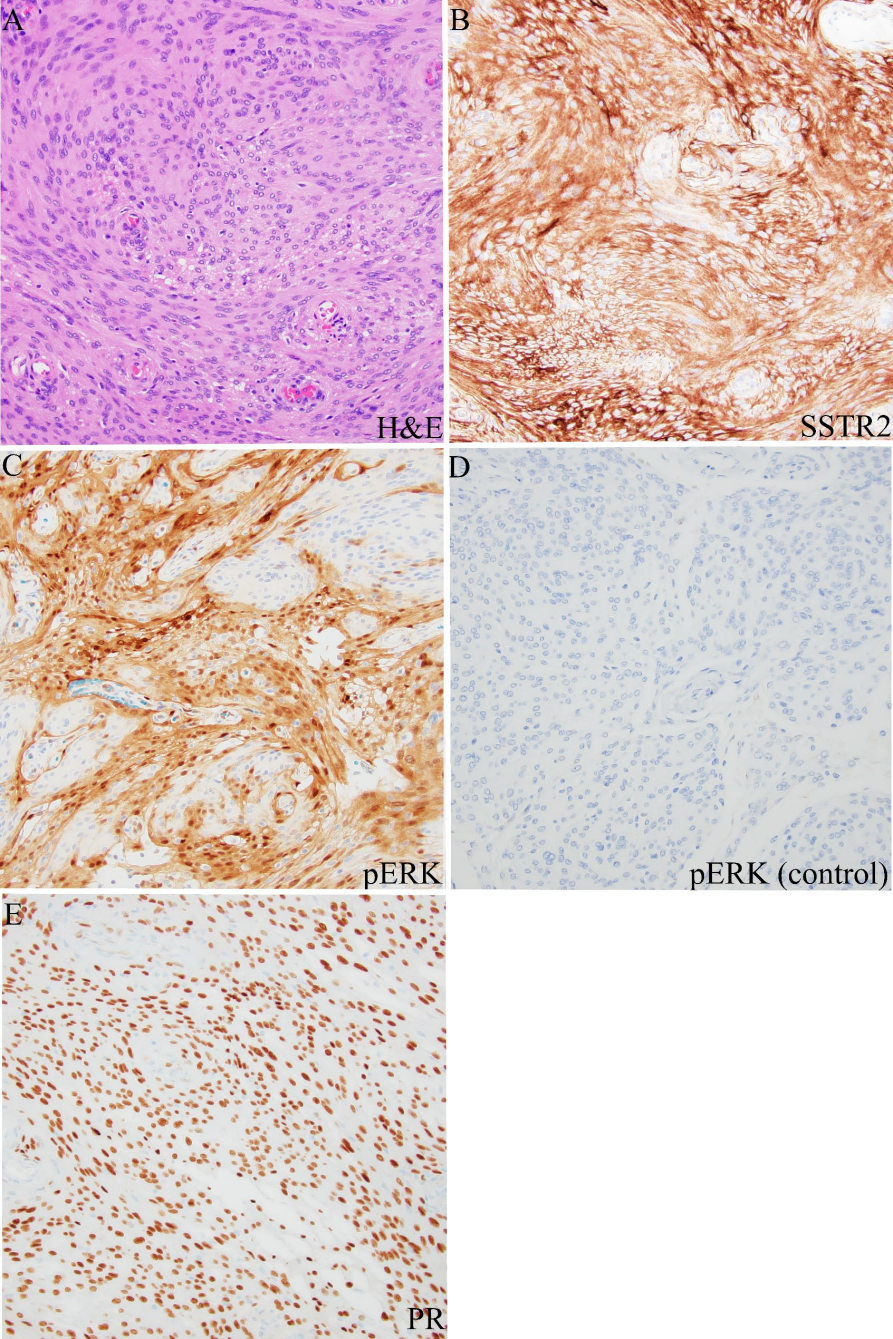
**A**) H&E stain of resected tumor showing oval to round bland nuclei with occasional nuclear pseudoinclusions arranged in whorls and fascicles. **B**) Immunohistochemical staining showing positive expression of somatostatin receptor 2a. **C**) pERK stain showing positive staining in a large subset of our patient’s tumor cells. **D**) Negative control pERK stain showing negative staining in a comparative WHO grade 1 meningioma. **E**) Immunohistochemical staining showing positive expression of progesterone receptor (PR). All photomicrographs taken at 200x magnification

**Fig. 3:**
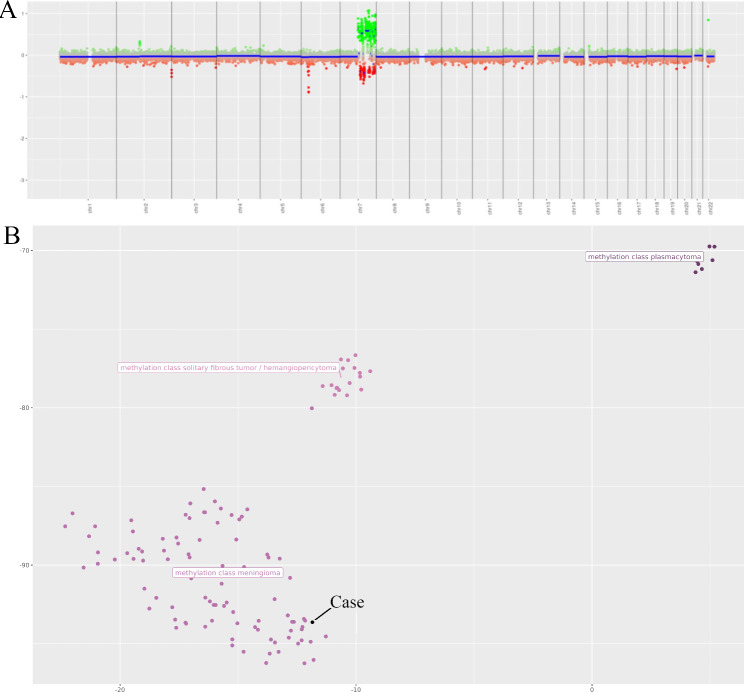
**A**) Copy number profile from DNA methylation sequencing reveals chromothripsis of chromosome 7. **B**) tSNE plot showing the current case clustering with meningioma

**Fig. 4:**
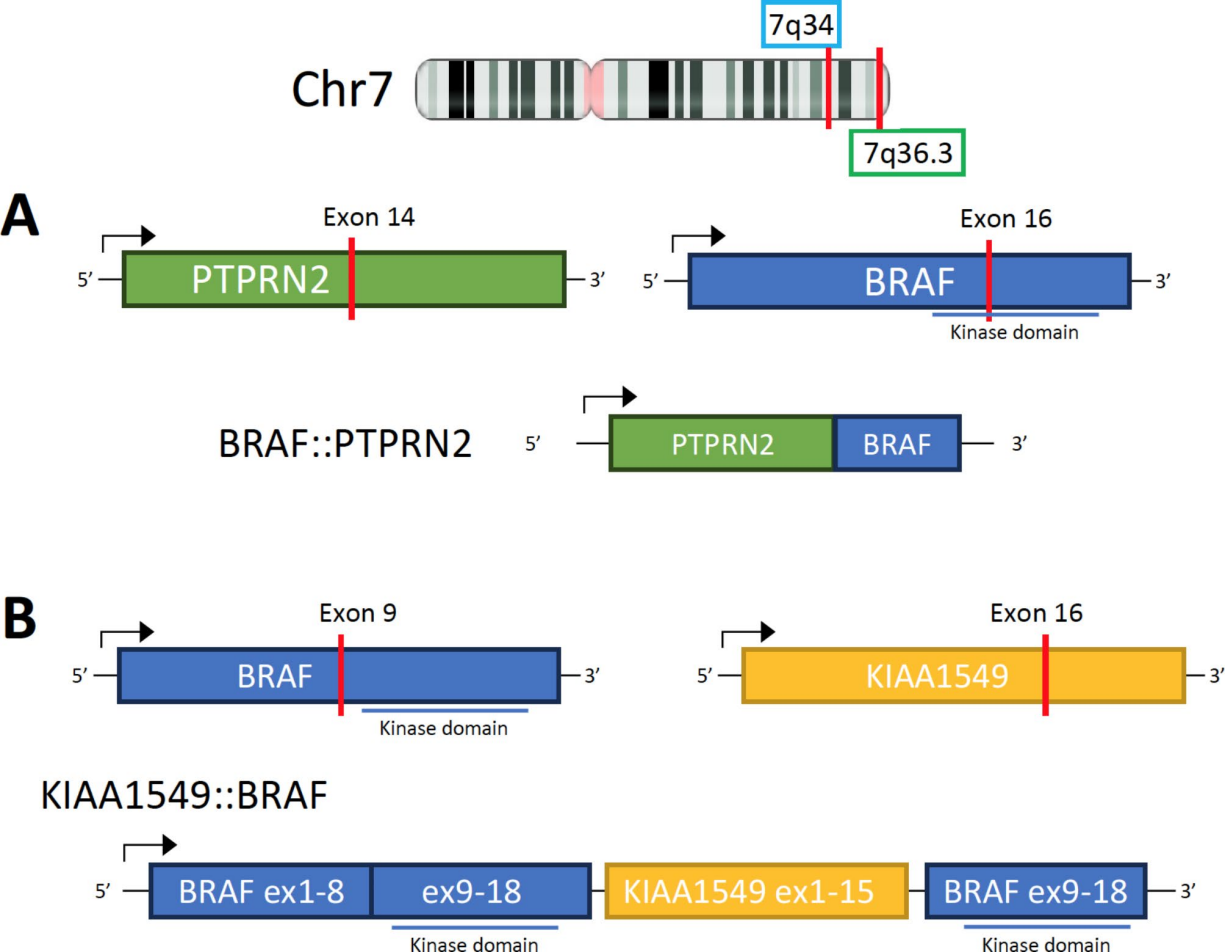
**A**) Gene fusion diagram showing BRAF gene on chromosome 7q34 and PTPRN2 gene on chromosome 7q36.3, resulting in an in-frame fusion between BRAF exon 16 and PTPRN2 exon 14, affecting the BRAF kinase domain. **B**) Comparative gene fusion diagram of a pilocytic astrocytoma fusion showing a BRAF::KIAA1549 gene fusion resulting from a duplication of BRAF on chromosome 7q34

Histopathology of the tumor sample showed an epithelioid neoplasm composed of oval to round, bland nuclei with occasional nuclear pseudoinclusions arranged in whorls and fascicles (Fig. 2a). Hypercellularity, microcystic features, and focal clear cell change were present. The sample was absent of necrosis, sheeting, prominent macro-nucleoli, and small cell change. There were no chordoid, papillary, or rhabdoid features present. Immunohistochemistry (IHC) stains for somatostain receptor 2a (SSTR2a) and progesterone receptor (PR) were positive in the tumor cells (Fig. 2b and e). Additionally, pERK staining of the sample showed strong positivity in a subset of the tumor cells, indicating increased *MAPK* activation (Fig. 2c). The DNA methylation profile was analyzed using the Illumina EPIC 850k platform, and matched with meningioma (confidence score 1.0). Post-hoc analysis revealed the tumor was in the Merlin-intact methylation group [12]. The sample was negative for *TERT* promoter mutation based on targeted sequencing. Standard workup of meningioma at our institution involves in-depth molecular analysis for recurrent tumors, WHO grades 2 or 3, and select WHO grade 1 tumors at the treating physician’s discretion. Due to the patient’s young age and very large size at presentation, molecular analysis was performed on her tumor to inform risk profiling and future treatments if necessary. The tumor sample was analyzed with PGDx Solid Tumor NGS panel, FusionPlex Solid Tumor NGS panel, and Oncoscan Solid Tumor Microarray. Interestingly, an in-frame fusion transcript *BRAF::PTPRN2* was detected along with chromothripsis of chromosome 7q, resulting in multiple segmental gains and losses, including amplifications of 7q21.2q22.1 (*CDK6*), 7q31.1q31.31 (*MET*), and 7q31.33q31.1 (*SMO*) (demonstrated based on DNA methylation profile in Fig. 3a). This *BRAF::PTPRN2* fusion is an in-frame fusion between *BRAF* Exon 16 on chromosome 7q34 and *PTPRN2* Exon 14 on chromosome 7q36.3, affecting the *BRAF* kinase domain **(**Fig. 4a**)**. Oncoscan also detected upregulation of the fused regions 7q34 (5 times copy number state) and 7q36.3 (8 times copy number state). The combined histologic, immunohistochemical, and molecular features supported the preoperative diagnosis of WHO grade 1 meningioma.

Postoperatively, the patient was transferred to the neurosurgery intensive care unit for recovery. The patient had mild diplopia, decreased sensation to pressure in the left V3 dermatome, and mild ptosis on the left, but was otherwise neurologically intact and doing well. The patient was discharged on postoperative day 2 and continued prophylactic anticoagulation therapy as well as continued Levetiracetam. At eight-week follow-up in clinic, the patient continued to do well with no further seizures, resolution of diplopia and improved facial sensation.

## Discussion and conclusions

The *BRAF* gene, located at chromosome 7q34, encodes the B-Raf proto-oncogene serine/threonine kinase, which regulates cell growth, division, and differentiation [27, 28]. Activating *BRAF* alterations can lead to a constitutively active B-Raf protein, resulting in neoplastic growth. The most common *BRAF* alteration is a V600E mutation, where the substitution of valine with glutamic acid at position 600 causes hyperactivation of the protein [29, 30]. Previous studies have reported different *BRAF* mutations in colorectal, lung, thyroid cancers, and melanoma [28, 31–33]. *BRAF* mutations in meningioma, though rare, have been reported in the literature with the majority being *BRAF* V600E point mutations in rhabdoid meningioma [34, 35].

Targeted therapies have been developed to inhibit constitutively activated *BRAF* proteins. *BRAF* inhibitors (BRAFi), such as vemurafenib and dabrafenib, have shown significant efficacy in targeted treatment of patients with *BRAF* positive cancers such as melanoma [36, 37]. BRAFi therapy may also have potential efficacy for treating *BRAF*-activated mutant primary brain tumors, such as gliomas and astrocytomas, based on initial data [38, 39]. However, resistance to BRAFi therapy remains a significant challenge in the treatment of *BRAF*-activated primary brain tumors [39–41].

*BRAF* gene fusions are a less common subtype of *BRAF* genetic alteration that have been reported primarily in brain tumors. Several studies have reported on the *BRAF::KIAA1549* fusion in pilocytic astrocytomas as well as diffuse leptomeningeal glioneuronal tumors (DLGNT) [42–44]. The resultant protein, containing the N-terminal region of *KIAA1549* and the kinase domain of *BRAF*, leads to activation of the *MAPK* signaling pathway and contributes to oncogenesis [45].

Given the availability of clinical inhibitors, *BRAF* alterations have been investigated as a potential treatment target in multiple cancers. However, to the best of our knowledge, the fusion of *BRAF* with *PTPRN2* in a WHO grade 1 meningioma has not been previously reported. *PTPRN2*, also found on chromosome 7, encodes the protein Islet Antigen-2β (IA-2β) which belongs to the protein tyrosine phosphatase (PTP) family and plays an important role in insulin secretion [46]. IA-2β may play a role in glucose intolerance in insulin-dependent diabetes [47]. However, the full activity of IA-2β has not yet been elucidated and its tyrosine phosphatase activity has not been experimentally validated. *PTPRN2* is upregulated in breast cancer and has been shown to increase tumor growth and metastatic potential in mouse models [48]. Additionally, epigenetic modification *PTPRN2* has been suggested to play a role in the oncogenesis of hepatocellular carcinoma [49], and it is hypomethylated in some glioblastomas, glioblastoma stem cells and primary xenografts [50]; however, it has never been reported to be altered in meningioma. In our patient, upregulation of the sites of *BRAF::PTPRN2* fusion at 7q34 and 7q36.3, along with positive pERK staining in tumor cells, raise the possibility that this novel fusion may contribute to oncogenesis by increased *MAPK* activation, but further experimental validation would be necessary to define the impact of the fusion.

Chromothripsis of chromosome 7q was also identified in our patient, resulting in amplifications of *CDK6, MET*, and *SMO*. Chromothripsis is a single, large event that involves complex chromosomal rearrangements, including deletions, duplications, inversions, and translocations resulting in reorganization of genes [51, 52]. While the frequency of chromothripsis in meningiomas is not well-established, cases have been reported [16, 53]. Some studies have suggested that cancers with complex chromosomal rearrangements, such as chromothripsis, may exhibit more aggressive behavior, progression to higher grade, increased recurrence rates, or altered response to treatment [51, 54, 55]. However, further research is needed to better understand these associations.

The identification of the *BRAF::PTPRN2* fusion in meningiomas is novel, and highlights the need for further interrogation of gene fusions in meningioma tumorigenesis and progression. While the clinical and biological implications of the *BRAF::PTPRN2* fusion are not fully known, further investigation could offer insight into the mechanisms of meningioma development and recurrence after surgical treatment. Further mechanistic and clinical research of this novel fusion is needed to determine its potential oncogenesis and whether patients with such fusions would be good candidates for anti-*BRAF* therapy.

In conclusion, we report a novel *BRAF::PTPRN2* fusion with associated chromothripsis of chromosome 7q in a case of a WHO grade 1 meningioma. These findings contribute to a growing literature of the genomic profiling in meningiomas. Further studies are needed to detail the clinical and pathological significance of *BRAF* fusions as well as to explore the potential of targeted therapies for meningioma.

## Data Availability

Not applicable.

## List of abbreviations

BRAF: V-raf murine sarcoma viral oncogene homolog B1
WHO: World Health Organization
PTPRN2: protein tyrosine phosphatase receptor type N2
CDK6: cyclin dependent kinase 6
MET: tyrosine protein kinase Met
SMO: smoothened
MAPK: mitogen-activated protein kinase
SRS: stereotactic radiosurgery
NGS: next generation sequencing
EEG: Electroencephalogram
MRI: magnetic resonance imaging
IHC: immunohistochemistry
BRAFi: BRAF inhibitor
DLGNT: diffuse leptomeningeal glioneuronal tumor
IA-2β: islet antigen-2β
PTP: protein tyrosine phosphatase
